# Some Unusual Symptoms in a Case of Hydrocephalus

**Published:** 1886-06

**Authors:** W. Barrett Roué

**Affiliations:** Physician Bristol Hospital for Sick Children and Women


### SOME UNUSUAL SYMPTOMS IN A CASE OF HYDROCEPHALUS. By W. Barrett Roué, M.D., M.S., F.R.A.S., Physician Bristol Hospital for Sick Children and Women.

E. M. S., a female child, 11 years of age, from Swindon, was admitted into the Children's Hospital and placed under my care on the 21st of March last.

She had fallen downstairs some five months previously, and had developed choreic movements a fortnight after the fall. There was no history of rheumatism, scarlet fever, or convulsions. Physical examination revealed nothing: all her organs appeared to be normal. She had, however, right internal strabismus, which had been present seven years, and was unaccounted for. Temp. and pulse normal. The child since her fall had complained of constant severe headache, which was present on her admission.

She was placed upon sedative treatment, with a view to subdue her headache and arrest the choreic movement.

April 4th. Headache better. Movements as before. Heart sounds normal.

April 15th. Much in the same condition. On this day, as the child's bowels were very sluggish, and the exhibition of aperients seemed to cause sickness, a simple enema was ordered.

April 16th. The child lies in a stupid, drowsy condition. Vomiting set in directly after the enema, as after the aperient medicine. Choreic movements continue.

Meanwhile the child had been taking liq. arsenicalis and ammon. citrate of iron as medicine, with ordinary diet, which, on the occurrence of the sickness, I thought it advisable to change for bismuth and hydrocyanic acid, with milk and beef tea.

The next note of importance is—

April 18th. Has not vomited since. Seems very drowsy, and does not notice anything that goes on around her. Pupils rather dilated, and respond but feebly to light.

April 20th. Vomited again yesterday. Child does not experience any previous nausea, but vomits suddenly, almost before she can reach the basin which stands upon the bed.

April 21st. This evening, at five o'clock, when sitting up to take her tea, the child suddenly complained of total loss of sight. She is unable to detect the presence of a lighted candle brought close to her eyes. Her pupils are widely dilated, and do not in the least respond to varying light. It appears that three days ago she mentioned to the nurse that the sight of the right eye was somewhat dim. There has been no faintness, nor is there any paralysis.

On the following day an ophthalmoscopic examination of the eyes was attempted, but was found impossible, on account of the continual choreic movements of the child's head and accompanying nystagmus.

April 28th. Vomited again this morning. Pupils continue widely dilated. Complains of constant frontal headache.

The child remained much in the same state until May 8th, when the House Surgeon's notes state that "the pupils are contracted," but still do not respond to varying light.

On May 11th the vomiting increased. There was great difficulty in swallowing; and in spite of nutrient enemata the child gradually sank, and died on the 4th of June, seven months and a half after the fall.

*Post-mortem.*—Body much emaciated. Rigor mortis but very slightly marked.

*Brain.*—On removing the skull-cap, the vessels of the duramater were seen to be much dilated and filled with blood, the whole brain appearing to be enlarged and distended.

On opening the dura mater, the visceral and parietal layers of the arachnoid were found to be adherent along the upper margins of the longitudinal fissure, which contained a considerable quantity of inflammatory lymph. There was no excess of fluid in the subarachnoid space.

On examining the base of the brain after its removal, the interpeduncular space was found to be obliterated; the area limited by the optic tracts in front, and the crura cerebri behind, contained simply a cyst formed of pia mater bulged out by fluid present in the third ventricle.

On cutting into the cerebrum, the brain substance was found to be softened and congested. The lateral ventricles were enormously distended with a dirty-coloured fluid; the septum lucidum was very thin, almost transparent; the choroid plexuses small and empty.

The cavity of the third ventricle was much enlarged, its floor being formed entirely by the pia mater, which bulged into the interpeduncular space; the parts usually forming the floor—viz., the tuber cinereum, infundibulum, pituitary body, corpora albicantia, and posterior perforated space—were altered in position or had disappeared.

The optic thalami forming the sides of the third ventricle were much compressed and unusually pale. The optic nerves were in an advanced state of softening.

The cerebellum, medulla, and pons seemed normal. There was not the least sign of tubercle.

*Lungs.*—Emphysematous throughout.

*Liver.*—Enlarged and congested.

*Kidneys.*—Congested.

Remaining organs normal.

Before endeavouring to understand the clinical aspects of this case, it may be as well to glance briefly at the two kinds of hydrocephalus (if two kinds there be) which affect children.

The general result of much discussion as to the pathology of this disease is to lead to the conclusion that it arises from a chronic form of inflammation in the lining membrane of the ventricles and choroid plexuses. This conclusion points equally to the hydrocephalus of infants and young children before the fontanelles are closed, and to that of older children after the bones of the head are united. Here, however, I think all similarity ceases. In its symptoms and in its course, the hydrocephalus of older children is entirely distinct from that which is observed in infants and young children: the diseases with which they may be confounded are different, and there are points of difference in the treatment.

"Pathologically, then, the relation between the two is one of resemblance; clinically it is one of contrast." "The cranial enlargement which renders the diagnosis comparatively easy in the one set of cases, is wanting in the other; and in the symptoms which *are* present there is scarcely anything to distinguish it from other chronic affections of the brain."

In this case the diagnosis was complicated by the occurrence of sudden and complete blindness, which suggested some such immediate cause as hæmorrhage into some portion of the optic tract and commissure, and certainly did not strengthen one's impressions in favour of simple hydrocephalus.

In the present state of our knowledge I must say I think the diagnosis between hydrocephalus in older children after the bones of the head are knit, and other cerebral affections, is often exceedingly difficult,—now and then impossible. In considering the terrible havoc made in this case by the presence of fluid in the ventricles,—chiefly, I am convinced, mechanical, the result of pressure,—some interesting questions suggest themselves.

Will hydrocephalus in older children and adults be in the future added to the rapidly-increasing list of diseases in the treatment of which the physician must stand aside for the surgeon ?

The great difficulty that at present stands in the way of such a procedure, is one of diagnosis.

Will our diagnostic powers so increase and improve that we shall be able to make out the presence of fluid in the ventricles of the brain as certainly as we can in the pleura, and call in surgical aid with as happy results ? I must say I am hopeful. I trust that physicians, by combined efforts in noting and registering the smallest variations in the symptoms between hydrocephalus and other chronic brain diseases, will eventually arrive at a diagnosis sufficiently certain to enable effusion into the ventricles to be treated on the general principles of surgery.

Exception has been taken to the above hopeful view of the operative treatment of hydrocephalus, on the ground that Mr. Hilton's view of the pathology of this disease is the generally accepted one. In the first place, I believe that the "medical mind" is rapidly changing its attitude in reference to this view; and in the second, I have yet to learn that the general acceptance of a doctrine is any *proof* of its truth. The late Dr. Hilton Fagge, at any rate, speaks on the subject with no uncertain voice. He says:

"Several pathologists have shown that there is sometimes closure by adhesions, either of the foramen at the lower angle of the fourth ventricle, or of the aqueduct of Sylvius. And Hilton propounded the doctrine that the obliteration of these channels is often the cause of hydrocephalus, by preventing the outflow of the secretion of the choroid plexuses into the subarachnoid space of the cord, which ought normally to occur whenever an increase in the physiological activity of the brain leads to an augmentation of its blood supply. But it is difficult to see why the fluid should continue to be poured out under the increased pressure which must necessarily result, and which certainly is present in most cases of hydrocephalus. And as the adhesions themselves are the *results* of a more or less widely diffused meningitis, such as is often attended with an inflammatory change in the ependyma, it would seem more reasonable to regard this as the *cause* of the effusion than to adopt Hilton's theory. It is well known that in one and the same case the various serous membranes may pour out fluids of very different specific gravity; and I think it is not improbable that the lining of the ventricles of the brain may continue to secrete a fluid containing scarcely any albumen, even when it is affected with a low degree of inflammation."*

* The italics in the quotation are mine.—*W.B.R.*

